# An Extremely Rare *SRD5A2* Gene c.485A>C Mutation in a Compound Heterozygous Newborn with Disorders of Sex Development First Identified in Vietnam

**DOI:** 10.1155/2022/6025916

**Published:** 2022-03-27

**Authors:** Phan Tuong Quynh Le, Thanh Nha Uyen Le, Thi Thanh Binh Nguyen, Minh Thao Nguyen, Thi Minh Thi Ha

**Affiliations:** ^1^Department of Medical Genetics, University of Medicine and Pharmacy, Hue University, Hue, Vietnam; ^2^Center of Prenatal and Neonatal Screening-Diagnosis, University of Medicine and Pharmacy Hospital, Hue University, Hue, Vietnam; ^3^Department of Pediatrics, University of Medicine and Pharmacy, Hue University, Hue, Vietnam; ^4^Department of Anatomy and Surgical Training, University of Medicine and Pharmacy, Hue University, Hue, Vietnam

## Abstract

*SRD5A2* (steroid 5-alpha-reductase 2) mutation, which impairs 5*α*-reductase-2 enzyme activity, is among the causes of 46,XY disorders of sex development (DSD). Here, we report a rare pathogenic mutation NM_000348.4:c.485A>C (NP_000339.2:p.His162Pro) of *SRD5A2* gene in a compound heterozygous state first identified in a Vietnamese newborn with 5*α*-reductase-2 enzyme deficiency. We also first submitted this rare mutation to ClinVar database (VCV000973099.1). The patient presented with hyperpigmented labia-majora-like bifid scrotum, clitoris-like phallus, perineoscrotal hypospadias, and blind-ending vagina. The other mutation NM_000348.4:c.680G>A (NP_000339.2:p.Arg227Gln) was reported previously. This compound heterozygous mutation was first detected by next-generation sequencing. By Sanger sequencing, we confirmed that the c.485A>C mutation was maternal inherited, whereas the c.680G>A mutation was paternal inherited. Up to date, this is the first report of this rare compound heterozygous state of *SRD5A2* c.485A>C and c.680G>A mutations in patients with 46,XY DSD generally as well as in Vietnamese population particularly and is also the second report in the world carrying the pathogenic mutation NM_000348.4:c.485A>C (NP_000339.2:p.His162Pro). Our finding has enriched the understanding of the spectrum of *SRD5A2* variants and phenotypic correlation in Asian patients with 46,XY DSD.

## 1. Introduction

The 5-alpha-reductase-2 (5*α*RD2) deficiency (OMIM 264600), first described in 1974 by Imperato-McGinley et al. [1] and Walsh et al. [2], is a rare 46,XY disorder of sex development (DSD). The affected patients might present a wide spectrum of variable clinical manifestations from completely phenotypic females to normal males [3]. Female external genitalia with clitoromegaly or microphallus at birth, various degrees of hypospadias, and unilateral/bilateral cryptorchidism are the most common features [4, 5].

The 5*α*RD2 enzyme is important for the conversion of testosterone to dihydrotestosterone (DHT), which is more biologically active [6]. Testosterone plays a key function in stimulating the Wolffian ducts during sexual differentiation, whereas DHT controls the virilization of external genitalia, urethra, and prostate during embryogenesis and mediates masculinization at puberty [7].

The *SRD5A2* gene (steroid 5-alpha-reductase 2, OMIM 607306) on chromosome 2 (2p23.1) encodes the 5*α*RD2 enzyme. To date, approximately 180 variants of *SRD5A2* gene have been identified, with missense and nonsense mutations being the most common (Human Gene Mutation Database). The NM_000348.4:c.680G>A mutation (NP_000339.2:p.Arg227Gln) was detected in three Vietnamese patients in a homozygous state [5, 8] and was one of the most frequent mutations in Asians [9]. In 2019, Cheng et al. first reported a novel *SRD5A2* mutation (NM_000348.4:c.485A>C) (NP_000339.2: p.His162Pro) in adult Chinese twins with 5*α*RD2 enzyme deficiency [10]. However, the authors have not yet submitted this variant to ClinVar database. Herein, we present the data of the world's second report, a Vietnamese newborn with 46,XY DSD, who possesses this rare mutation in a heterozygous compound state. We also submitted this rare variant to ClinVar database for the first time (VCV000973099.1).

## 2. Case Presentation

**Figure 1:**
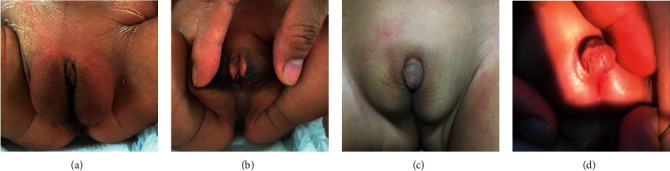
Patient's abnormal genital phenotype: at the age of three days, (a) labia-majora-like bifid scrotum with clitoris-like microphallus and (b) perineoscrotal hypospadias; at the age of two years and three months, (c, d) microphallus had grown to 1.5 cm in length.

The patient is the second child of healthy and nonconsanguineous parents, born at the 40^th^ week of gestation. The patient's external genitalia were found to be abnormal at birth. At the age of three days (November, 2019), physical examination showed a hyperpigmented labia-majora-like bifid scrotum with bilateral masses palpable (Figure 1(a)), a microphallus of 0.8 cm with clitoris-like underdeveloped glans, and perineoscrotal hypospadias with short blind-ending vagina (Figure 1(b)). At the age of two years and three months, a second medical examination revealed that the microphallus had grown to 1.5 cm in length (Figures 1(c), and 1(d)) and that the depth of blind-ending vagina was 1 cm (March, 2022). The patient was reared as a female at birth, and the gender was changed from female to male after being diagnosed. There was no history of DSD in the family. The patient's older sister, who is five years old, is developing normally.

Ultrasonography at newborn revealed the absence of Mullerian remnants and the presence of bilateral testes in the labia majora. The right testis was 11.4 × 7 × 6 mm in size, while the left was 11.5 × 9 × 6 mm in size. The rete testis and epididymis were both present. The structures of male urethra were observed. The penis with corpus cavernosa and corpus spongiosum was buried.

The blood samples' biochemical analysis revealed a normal baseline testosterone level of 1.04 ng/mL. There was no DHT level available.

**Figure 2:**
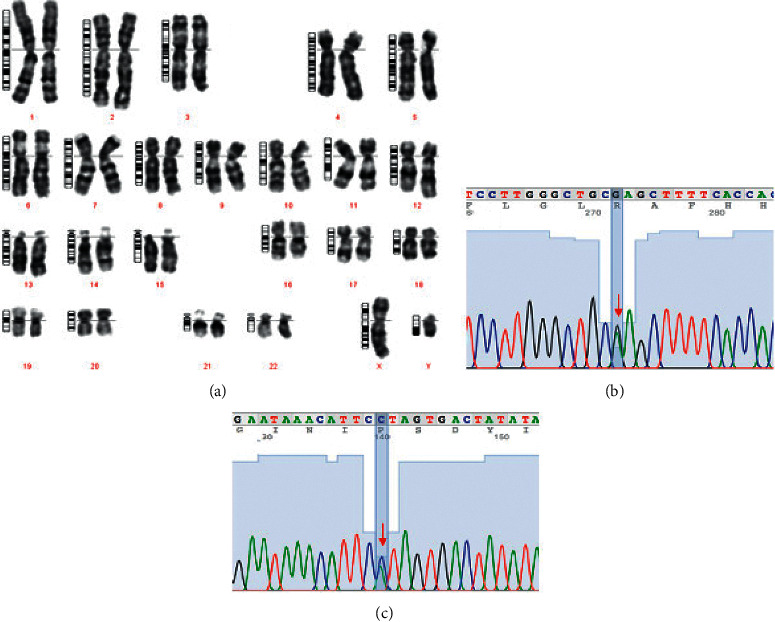
Genetic analysis of the patient. (a) 46,XY karyotype and (b, c) sanger sequencing results revealed c.680G>A (p.Arg227Gln) and c.485A>C (p. His162Pro) mutations.

The karyotype of the patient was 46,XY (Figure 2(a)). DNA was extracted from peripheral blood sample. Polymerase chain reaction (PCR) assay with *SRY*-specific primers revealed a result of positive *SRY*. DNA sample was subjected to next-generation sequencing (NGS) (NextSeq, Illumina, USA) using a panel of DSD related genes. A compound heterozygous for a rare mutation NM_000348.4:c.485A>C (NP_000339.2:p.His162Pro) and one well-known mutation NM_000348.4:c.680G>A (NP_000339.2:p.Arg227Gln) which located on exon 3 and exon 4 of *SRD5A2* gene, respectively, were found. These two mutations were further confirmed by Sanger sequencing (Figures 2(b) and 2(c)). We submitted the NM_000348.4:c.485A>C (NP_000339.2:p.His162Pro) mutation to ClinVar database on July 14, 2020. The accession number of this mutation is VCV000973099.1 (SCV001422495.1). Further evaluation demonstrated that the mutant c.485A>C was maternal inherited, whereas c.680G>A was paternal inherited. The patient's sister was healthy. She had neither c.485A>C nor c.680G>A mutations.

Written informed consent was obtained from the parents of the patient in accordance with the Institutional Review Board of University of Medicine and Pharmacy, Hue University, Vietnam.

## 3. Discussion

The patient presented bilateral testes inside the hyperpigmented labia-majora-like bifid scrotum, clitoris-like phallus, perineoscrotal hypospadias, and blind-ending vagina. The primary genetic analysis revealed the 46, XY karyotype and the presence of *SRY* gene. As a result of these findings, the patient was classified as 46,XY DSD. Although patient's symptoms have been reported frequently in 5*α*RD2 deficiency, this phenotype in a newborn has a high overlap with other common causes of 46,XY DSD related to androgen synthesis or function, such as partial androgen insensitivity and 17-hydroxysteroid dehydrogenase deficiency [11–13]. Hormonal analysis is a valuable diagnostic tool for patients with 46,XY DSD. In newborns, the baseline testosterone level in a blood sample can indicate the ability of the testes to secrete testosterone [14]. However, this patient has a normal testosterone level, which can be found in both androgen insensitivity syndrome and 5*α*RD2 deficiency.

Several genes responsible for 46,XY DSD have been identified. As a result, sequencing a panel of those candidate genes is first recommended when clinical and hormonal patterns are insufficiency to indicate a diagnosis [14]. The compound heterozygous status for c.485A>C (p.His162Pro) and c.680G>A (p.Arg227Gln) of the *SRD5A2* gene, which was identified via NGS, confirmed 5*α*RD2 deficiency in our patient.

To date, around 180 mutations have been described in the *SRD5A2* gene, among which missense mutations are major (Human Gene Mutation Database, HGMD, https://www.hgmd.cf.ac.uk/ac/gene.php?gene=SRD5A2). Our patient carries a rare compound heterozygous mutation of *SRD5A2* gene, including maternal inherited c.485A>C in exon 3 and paternal inherited c.680G>A in exon 4. Interestingly, exon 1 and exon 4 of the *SRD5A2* gene are known as hotspot regions, whereas the frequency of mutation happening in exon 3 is quite rare [15, 16]. This is the first report in the world of a compound heterozygous state of SRD5A2 c.485A>C and c.680G>A mutations.

The *SRD5A2* c.485A>C mutation is an extremely rare variant. In 2019, Chinese 18-year-old twin brothers with DSD (sex of rearing at birth changed from female to male) possessing a compound heterozygous state of *SRD5A2* c.485A>C (p.His162Pro) and c.16C>T (p.Glu6Ter) mutations were first reported [10]. The parental origin of these variants was not identified in Cheng et al.'s study. Our case is the second report of c.485A>C mutation in the world. We submitted the c.485A>C mutation in exon 3 of the *SRD5A2* gene to ClinVar database to enrich the variant data of this gene.

The homozygous c.680G>A (p.Arg227Gln) mutation was reported previously in three Vietnamese patients with 5*α*RD2 enzyme deficiency [5, 8]. However, they were all diagnosed outside of Vietnam. This mutation was also detected in a number of Asian patients with 5*α*RD2 deficiency, including those from China, Japan, Laos, and Mongolia [5]. In particular, Fan showed that c.680G>A (p.Arg227Gln) was the most common (39.62%) in 130 Chinese children with this disorder [9].

*SRD5A2* mutations can result in either a total loss of activity, disrupting the binding domain to testosterone, or an inefficient protein assembly or a shorter half-life [4]. The c.680G>A (p.Arg227Gln) and c.485A>C (p.His162Pro) mutations have been well documented as decreasing the 5*α*RD2 enzyme activity by functional analysis [10, 17]. Thus, carrying these two mutations, which may result in a significant loss of enzyme activity, contributed to our patient's abnormal phenotype. According to Fan, the group with the p.Arg227Gln mutation had milder and more variable characteristics than the group without this mutation. In particular, individuals with homozygous p.Arg227Gln mutations had minor undervirilization, whereas patients with compound heterozygous p.Arg227Gln mutations had varying degrees of undervirilization depending on the combination of mutations [9]. Previously reported Vietnamese patients with the homozygous p.Arg227Gln mutation had a microphallus, bifid scrotum with testes inside; hypospadias was recorded in two out of three [5, 8]. Our patient, who was a compound heterozygote for p.Arg227Gln and p.His162Pro, was more undervirilized than Vietnamse patients with homozygous p.Arg227Gln but less undervirilized than twin brothers with the compound heterozygous p.His162Pro and p.Glu6Ter mutations reported by Cheng et al. (female external genitalia, clitoromegaly, and gonads bilateral in inguinal position) [10].

As mentioned above, the patient's phenotypic features, such as bilateral testes in scrotum, a microphallus with clitoris-like underdeveloped glans at birth which had grown into a small penis with 1.5 cm in length at the age of 27 months, and male urethral structures and the absence of Mullerian remnants determined via ultrasound, all lean towards a male phenotype. Furthermore, the molecular diagnostics confirmed 5*α*RD2 deficiency in our patient. As a result, the patient was reared as a male. Psychosexuality is one of the most attentive considerations in 5*α*RD2 deficiency individuals. Even in the absence of dihydrotestosterone, the presence of androgens usually directs towards male psychosexuality development. This could explain the relatively high rate of gender change from female to male in 5*α*RD2 deficiency, estimated 56–63% [18, 19]. Because our patient was diagnosed early and was raised as a male, the conflict in identifying sex will be avoided.

Surgical repair for hypospadias is recommended, mainly focusing on chordee correction and urethral reconstruction. Orchidopexy is unnecessary as the bilateral testes are in correct position. Surgery can be performed in two or three steps due to perineoscrotal hypospadias [4]. The location of the patient's testes in the scrotum, as well as the early correction of hypospadias, will help to preserve testicular function, particularly spermatogenesis, for future fertility [20].

Hormone therapy is an important aspect of management for some types of 46,XY DSD. Most male patients with 5*α*RD2 deficiency do not require the testosterone replacement because of retained testicular function during puberty [4]. However, testosterone treatment may help our patient, who has a micropenis, gain a benefit in terms of growth [21].

In addition to the patient's treatment, genetic counseling is required for parents who plan to have another child. As the parents are carriers, prenatal diagnosis should be mentioned in the next pregnancy. In vitro fertilization with preimplantation genetic testing will also be an option.

In conclusion, this is the first report of the extremely rare mutation NM_000348.4:c.485A>C (NP_000339.2:p.His162Pro) of the *SRD5A2* gene in a compound heterozygous state in patients with 46,XY DSD generally as well as in Vietnamese population particularly. This finding has enriched the understanding of the spectrum of *SRD5A2* variants and phenotypic correlation in Asian patients with 46,XY DSD.

## Data Availability

The NM_000348.4:c.485A>C (NP_000339.2:p.His162Pro) mutation of SRD5A2 gene was already submitted to ClinVar database, accession number VCV000973099.1 (SCV001422495.1).
